# Impact of radiotherapy on circulating lymphocyte subsets in patients with esophageal cancer

**DOI:** 10.1097/MD.0000000000020993

**Published:** 2020-09-04

**Authors:** Yajuan Lv, Meijuan Song, Xiufang Tian, Xinshuang Yv, Ning Liang, Jiandong Zhang

**Affiliations:** a Shandong Provincial Qianfoshan Hospital, Shandong University; b Department of Oncology, The First Affiliated Hospital of Shandong First Medical University, Jinan, Shandong, China.

**Keywords:** esophageal cancer, immunity, lymphocyte subsets, radiotherapy

## Abstract

Radiotherapy (RT) can affect the immune function of patients with cancer. The purpose of this study was to investigate the effect of RT on lymphocyte and its subsets in patients with esophageal cancer (EC).

All patients received RT with a mean dose of 5369 cGy (gray). Blood parameters were measured in 31 patients on 3 occasions (before, at the end of radiotherapy, and at 3 months follow-up). The whole blood count and lymphocyte subsets were measured and correlated with short time efficiency and radiation dose parameters.

White blood count (WBC) and lymphocyte count (ALC) were greatly decreased at the end of radiotherapy, and the percentages of CD3+, CD3+CD8+ T cells were significantly increased, on the other hand, a decrease in the CD4/CD8 ratio was observed. The percentages of CD3-CD16/56+NK cells and CD19+ B cell were decreased at the end of RT compared with prior RT. The percentages of CD3+ T cells before RT and the WBC and ALC count after RT can be used as prognostic indicators for survival. The PTV dose can cause significant changes in lymphocytes count after RT. CD3+T cells after RT were significantly correlated with mean heart dose and heart V50.

Our study identified that RT causes changes in lymphocyte subsets, and these changes may indicate differences in immune function between individuals. Radiotherapy plan should be designed to minimize normal tissue dose to reduce the impact on WBC and lymphocytes.

## 1 Introduction

Esophageal cancer (EC) is the sixth-leading cause of cancer-related deaths worldwide.^[1]^ The incidence of EC is very high in China, where most patients are diagnosed at advanced stage and the operation is not applicable.^[2]^ The prognosis of patients with cancer depends on the methods of treatment and the state of the cancer itself, but other factors, including immune defenses against cancer, also have an important impact on the survival. Patients with EC usually have a poorer nutritional state because of dysphagia than patients with other cancers. Thus, patients with EC are in a poorer immunological state than patients with other carcinomas. The immunity is believed to play a central role in cancer suppression. Chemoradiation (CRT) is widely accepted as the standard treatment in patients with advanced EC.^[3]^ Radiation-associated lymphopenia has been explored in a variety of malignancies.^[4,5]^ The major side effect of CRT is immunosuppression, caused by radiation exposure and anti-cancer drugs, which decreases the functions of leukocytes.^[4]^ Lymphocytes, one of the most radiation sensitive cell, account for approximately 30% of the normal white blood cell (WBC) population and are essential effector cells in anti-tumor immunity.^[6]^

Patients undergo severe treatments, such as radiotherapy (RT) and chemotherapy, and these treatments may further depress the immunity of the patients.^[7,8]^ Recent researches showed that RT induces severe lymphopenia in a range of cancers, and lymphopenia is associated with tumor progression and survival.^[9,10]^ And this may be correlated with prognosis. Many studies had reported the correlation between immunity and prognosis for lung and esophageal cancer.^[11,12]^ These findings strongly suggest the importance of immunity in the prognosis of cancer. A study demonstrated that a higher absolute lymphocyte count (ALC) level during neoadjuvant chemoradiation (nCRT) is associated with a higher rate of complete pathological response (pCR) for EC patients.^[13]^ Patients with pretreatment lymphopenia had a lower overall response rate(ORR) to chemotherapy than that of patients without pretreatment lymphopenia.^[14]^ Recent a study showed that the percentages of lymphocyte subsets differed between pre-RT and post-RT in the EC patients. Three months after RT, the percentages of CD3+, CD4+, and CD8+ T cells, and NK cells had recovered to the level before RT.^[8]^

In this study, we investigated the effect of RT on lymphocyte subsets in EC patients and also discussed the correlation between lymphocyte subsets and survival. In addition, the dosimetric parameters that may induce changes in WBC and lymphocyte subsets have also studied.

## 2 Methods and materials

### 2.1 Patients and clinical data

A total of 31 patients with EC were enrolled in this study. All the patients met the following inclusion criteria: first pathologically diagnosed with esophageal squamous cell carcinoma (ESCC); aged from 18 to 80 years old; KPS ≥70; no distant metastasis. The exclusion criteria included: prior chemo-radiotherapy or surgery; history of another primary cancer; the presence of hematologic disorders or inflammatory or autoimmune diseases; serious medical diseases that may affect survival. All patients provided informed consent. We have obtained consent from ethics committee of our hospital. We obtained patient characteristics from electronic records. Peripheral blood parameters were measured in all patients on 3 occasions (before RT, at the end of RT, and at 3 months follow-up) named T1, T2, T3, respectively. The short-term clinical efficiency was assessed at the end of RT according to the Response Evaluation Criteria in Solid Tumors (RECIST): complete remission (CR), partial response (PR), stable disease (SD), and progressive disease (PD). Effective response (ORR) defined as (CR+PR). Ineffective response defined as (PD+SD).

### 2.2 Radiotherapy procedure

The Eclipse 10 planning system (Varian Medical Systems, Palo Alto, CA) was used for treatment planning and dose distribution calculations. Intensity-modulated radiation therapy (IMRT) plans were created for each patient. The prescription dose was 45 to 60 Gy (gray) (once per day, 1.8–2.0 Gy each time). Dosimetric parameters like PTV volume, PTV dose, heart V5, V10, V20, V30, V40, V50, mean heart dose, mean body dose, mean bone dose, spleenV5, V10, V20, V30 were collected from DVH map. Vx was the volume that was irradiated above a designated dose.

### 2.3 Flow cytometric immunophenotyping

The proportion of lymphocyte subsets in peripheral blood were measured using flow cytometry at 3 times point (T1, T2, T3).

### 2.4 Statistical analysis

Data for continuous variables were expressed as mean ± SD. Dynamic change of parameters on 3 time points between-group were compared using independent *t* test and one-way ANOVA. Pearson correlation analysis was applied to analyze the correlation between dose parameters and variation in lymphocyte subset counts. Receiver operating characteristic (ROC) curve analysis was used to evaluate the sensitivity and specificity of the blood variables for predicting overall survival (OS). All *P*-values were 2-sided, and *P* values <.05 were considered significant. Statistical analyses were performed using GraphPad Prism Version 6.07 and SPSS (IBM) Version 22.

## 3 Results

### 3.1 Patient characteristics

The characteristics of all the patients are shown in Table 1. There were 25 men and 6 women with mean age of 61 years at the time of diagnosis. There 3 cases were in stage I, 9 were in stage II, and 19 were in stage III according to the AJCC 7th clinical stage of esophageal cancer. A total of 20 cases received RT and concurrent chemotherapy. There were 11 cases received only RT.

**Table 1:**
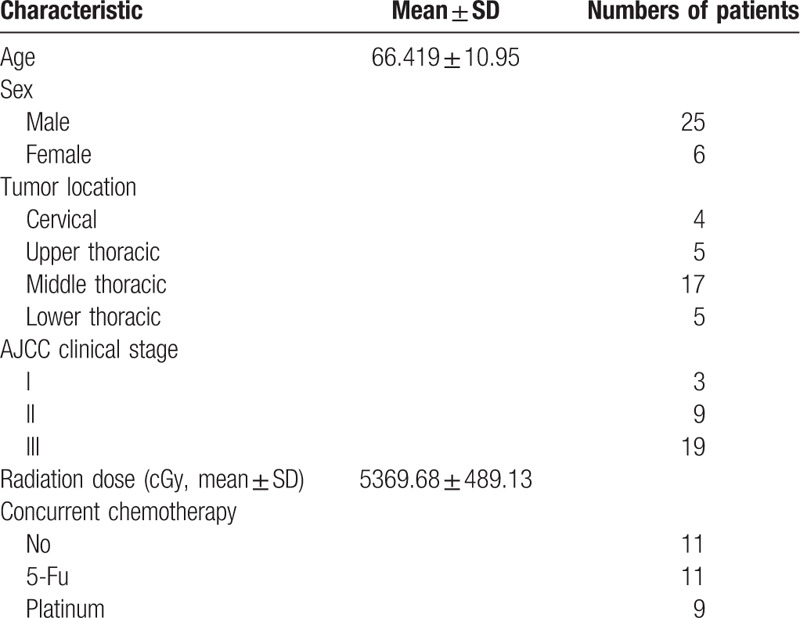
Patient characteristics (n = 31).

### 3.2 Dynamic change of parameters over time

**Figure 1:**
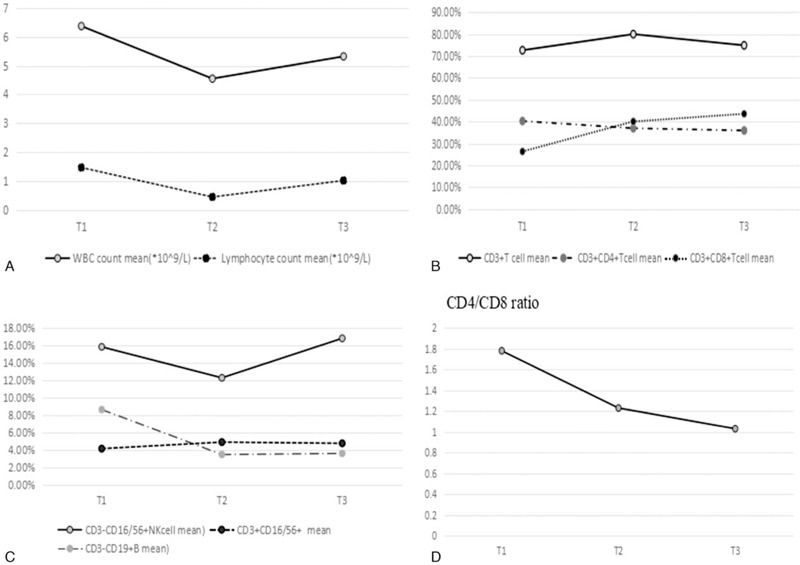
Variations in peripheral blood indexes for 3 time points. A. Variations in WBC, total lymphocyte cells count for 3 time points. B. Variations in CD3+, CD4+, CD8+T cells for 3 time points. C. Variations in NK and B cells for 3 time points. D. Variations in CD4/CD8 ratio for 3 time points. Data are present as mean value. T1: pre-RT. T2: the day when the full course of radiotherapy was completed. T3: 3 months after the final fraction of RT. RT = radiotherapy; SD = stable disease; WBC = white blood count.

As shown in Fig. 1 and Table 2, WBC count and ALC were greatly decreased at the end of radiotherapy, and the percentages of CD3+, CD3+CD8+ T cells were significantly increased, on the other hand, a decrease in the CD4/CD8 ratio was observed. The percentages of CD3-CD16/56+NK cells and CD19+ B cell were decreased at the end of RT compared with prior RT (*P* < .05). At 3 months after RT, the WBC count and percentages of CD3+T cells were recovered to the level before RT. But the ALC and the percentages of CD3+CD8+, CD3-CD19+ cells, and CD4/CD8 ratio were not recovered to the level before radiotherapy (Table 2).

**Table 2:**
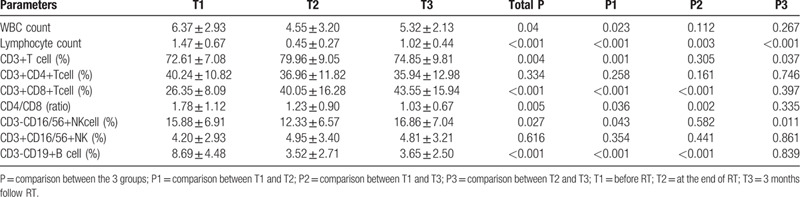
Dynamic change of parameters over time.

### 3.3 The relationship between immune parameters and short-term efficiency

**Figure 2:**
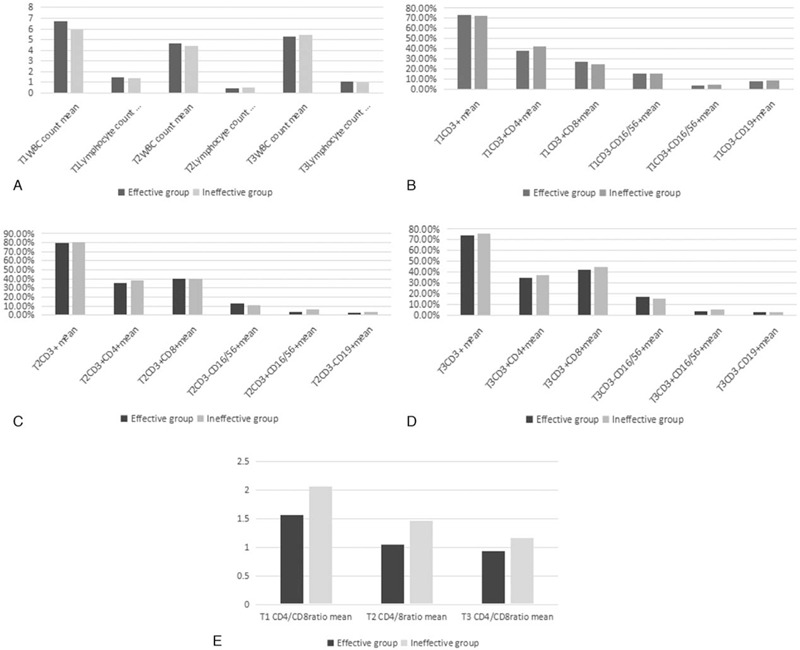
Variations in blood subsets in effective (CR+PR) and ineffective (SD+PD) group for 3 time points. A. WBC and ALC count comparisons between effective response and ineffective groups for 3 time points. B. CD3+, CD4+, CD8+, CD16/56+, CD19+ comparisons between effective and ineffective response groups at T1. C. CD3+, CD4+, CD8+, CD16/56+, CD19+ comparisons between effective and ineffective response groups at T2. D. CD3+, CD4+, CD8+, CD16/56+, CD19+ comparisons between effective and ineffective response groups at T3. E. The CD4/CD8 ratio comparisons between effective and ineffective response groups for 3 time points. Data are present as mean value. ALC = lymphocyte count; CR = complete remission; PD = progressive disease; PR = partial response; RT = radiotherapy; SD = stable disease; T1 = pre-RT; T2 = the day when the full course of radiotherapy was completed; T3 = 3 months after the final fraction of RT; WBC = white blood count.

The short-term efficiency was assessed using RECIST criteria. Our study found that there were no significant differences in blood cell subsets between the effective and ineffective groups at 3 time points. (Fig. 2, *P* > .05).

### 3.4 ROC curve for the prediction of survival

**Figure 3:**
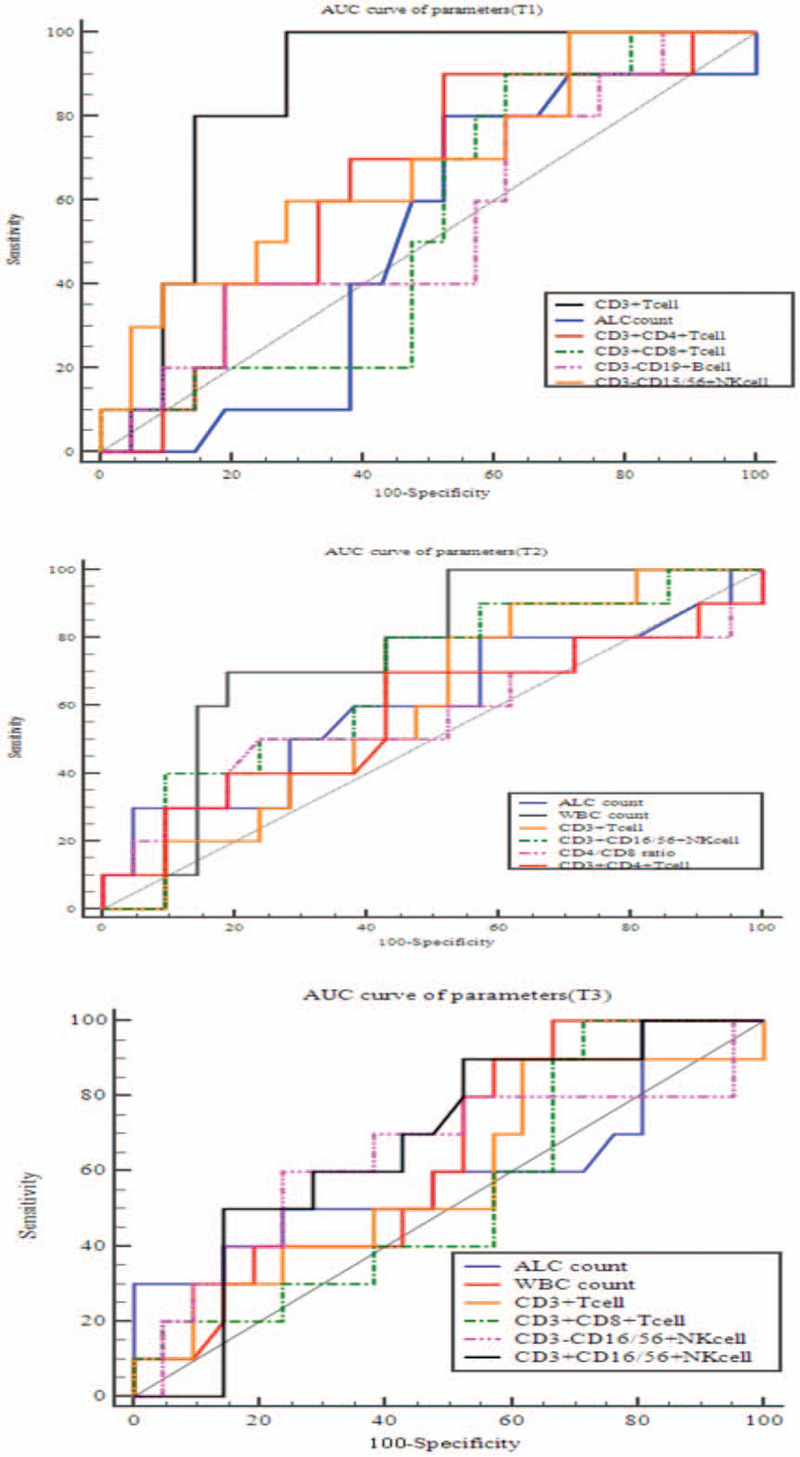
AUC curve of blood subsets for survival at 3 time points (T1, T2, T3). RT = radiotherapy; T1 = pre-RT; T2 = the day when the full course of radiotherapy was completed; T3 = 3 months after the final fraction of RT.

As shown in Fig. 3 and Table 3, the percentages of CD3+ T lymphocyte cells before RT can be used as a prognostic indicator for survival with AUC area 0.852 (sensitivity100, specificity 71.43, *P* < .001). WBC count and lymphocyte count at the end of RT were also prognostic indicators (*P* < .05).

**Table 3:**
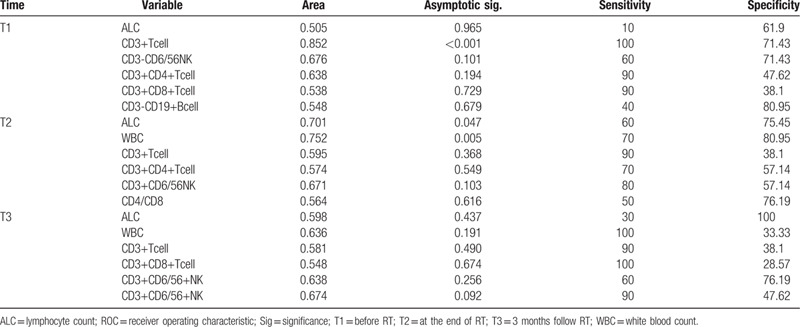
ROC curve for the prediction of survival.

### 3.5 Variations in lymphocyte subsets correlated with dose–volume parameters

**Figure 4:**
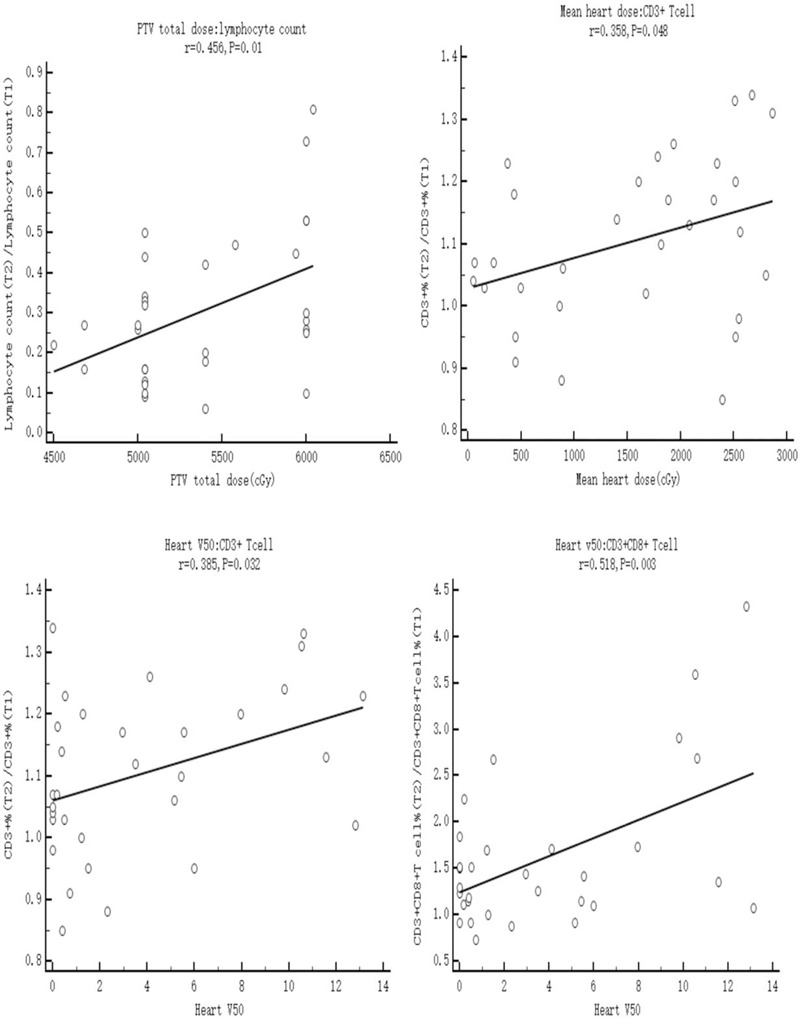
Person analysis of dose parameters and blood subsets at the end of RT (T2), assuming blood values at pre-radiotherapy (T1) of 1. A. Presents the relationship between total ALC count and PTV total dose (*P* = .01). B. Presents the relationship between mean heart dose and CD3+T cells (*P* = .048). C. Presents the relationship between heart V50 and CD3+ cells (*P* = 0.032). D. Presents the relationship between heart V50 and CD3+CD8+T cells (*P* = .003). ALC = lymphocyte count, RT = radiotherapy; T1 = pre-RT; T2 = the day when the full course of radiotherapy was completed; T3 = 3 months after the final fraction of RT.

Pearson analysis was used to compare the the correlations of lymphocyte subsets and dose–volume parameters. We compared the variations of all lymphocyte subsets in each patient with the dose–volume parameters (demonstrated in the method part above). We assumed blood values at pre-radiotherapy (T1) of 1. The PTV total dose can cause significant changes in lymphocytes count after RT (Fig. 4). CD3+T cells variation were significantly correlated with mean heart dose and heart V50 (*P* < .05). CD3+CD8+T cells were significantly correlated with heart V50 (*P* = .003) (Fig. 4).

## 4 Discussion

Patients with same cancer and receive the same treatment may have various outcomes. This may be caused by differences in individual immunity. The lymphocyte subsets and the natural killer (NK) cells are the immune cells most essential for the immunity of patients.^[15]^

The immune system plays a complementary role in the activity of RT.^[6]^ Patients undergoing CRT may have deficiencies in immunity.^[16]^ Cytotoxic drugs or radiation can release the tumor antigens through necrotic tumor cell death, relieve the immunosuppression mechanism.^[5,17]^ Poor prognosis was observed in EC patients with low lymphocyte percentage after CRT.^[18]^ RT caused significant lymphopenia compared with baseline.^[19]^ Lymphopenia may be associated with poor outcomes in cancer patients treated with RT.^[20]^ A study showed that pretreatment lymphopenia is an independent prognostic factor for patients with EC.^[14]^ Our study showed that WBC and ALC at the end of RT were prognostic indicators for survival (Fig. 3 and Table 3).

The WBC was increased in the RT group when compared with that in the chemotherapy or CRT groups, while the absolute counts of CD3+, CD4+, and CD8+ were relatively lower.^[16]^ While most of the focus in cancer immunity is on CD8+ cytotoxic T cells, recent study indicates that CD4+ T cells play an important role in the modulation of immune responses by enhancement and suppression of CD8+ responses.^[21]^ Successful immunity to cancer still require activation of CD4+ T cells.^[22]^ Previous reports have shown that CD4+ T cells play an anti-tumor role by enhancing cellular immune responses.^[23,24]^ A decrease of CD4+ T cells has been associated with poor prognosis in advanced cancer.^[25]^ A study reported that the decreased CD4/CD8 ratio was significantly associated with the poorer prognosis of patients with cervical cancer.^[26]^ Previous reports have shown that the ratio of CD4/CD8 reflects the immune status, and may predict mortality.^[27]^ The CD4/CD8 ratio was an independently prognostic factor for distant metastasis-free survival of patients with nasopharyngeal carcinoma.^[27]^ Wang et al^[8]^ showed that the percentages of CD4+ T cells, NK cells, and the CD4+/CD8 + ratio in EC patients before RT were significantly decreased but the percentage of CD8+ was significantly increased when compared with the healthy control. This study also demonstrated that the percentages of CD3+, CD4+ T cells, and NK cells and the CD4+/CD8+ ratio after RT were significantly reduced, but the percentage of CD8+ T cells was significantly increased, when compared with before RT. Three months after RT, the immune parameters had recovered to the level before RT.^[8]^ We found RT can cause significant changes in lymphatic subsets (Table 2). We found that the percentages of CD3+ T cells before RT can be used as a prognostic indicator for survival (Table 3).

Due to the fact that the lymphocytes plays a crucial role in immunity, especially anti-tumor immunity, the percentage of circulating lymphocyte subsets may be prognostic factors of efficiency. A study showed that a low density of CD8+ T cells after CRT was similarly associated with a poor response.^[28]^ Fang et al^[13]^ showed that EC patients receiving neoadjuvant CRT with high ALC nadir had a higher pathological response rate. The percentages of CD3+, CD4+ T cells, and NK cells, and the CD4/CD8 ratio of effective group (CR+PR) were markedly decreased compared with the ineffective radiotherapy group (SD+PD).^[8]^ The ORR in cancer patients with pretreatment lymphopenia was significantly lower than in patients with normal lymphocyte counts.^[29]^ In our study, we found that there were no significant differences in blood cell subsets between the effective and ineffective groups at three time points (Fig. 2). The reason may be the small sample size.

Multiple courses of RT, RT sites, and RT doses increased the risk of RT-associated lymphopenia.^[20]^ High RT doses were found to be a significant risk factor for lymphopenia in lung cancer patients.^[20]^ Wang et al^[8]^ showed that the immune parameters (percentages of CD3+, CD4+ T cells, and NK cell) were dramatically decreased after RT in the PTV volumes >300 cm^3^ group, whereas the percentage of CD8+ T cells increased, when compared with before RT. The paper indicated that the RT total dose did not have a significant effect on the immune parameters after RT compared with before RT. A study noted that the tumor volume irradiated was significantly associated with a decrease in ALC.^[30]^ A recent study reported that compared with those receiving RT to the brain, or abdomen, declines in the ALC were greater in those receiving RT to the spine, lung, and chest wall.^[31]^ A predictor of high ALC in EC patients is mean body dose.^[13]^ PTV volume was significantly correlated with lymphopenia.^[16]^ Prior studies demonstrated that in patients with lung cancer who underwent RT, the volume of lung receiving 5 Gy (V5) was significantly correlated with lymphocyte count.^[30]^ CD8+ T cells were found to be the most sensitive to radiation-induced apoptosis.^[32]^ However, the dose–response correlations has not been clear. One of the possible reasons is that the lymphocytes are highly radio-sensitive. A recent study showed that predictors of grade 4 (G4) ALC nadir included distal tumor location, definitive CRT, mean body dose, and chemotherapy. Tumor location in the lower third of the esophagus versus mid/upper esophagus was positively associated with G4 ALC nadir.^[33]^ Our study showed that the PTV total dose was significant correlated with lymphocytes count after RT. CD3+T cells were significantly correlated with mean heart dose and heart V50 (*P* < .05). CD3+CD8+T cells were significantly correlated with heart V50 (Fig. 4).

There were several limitations in our study. It was a single-institution experience. Radiation dose and chemotherapy drugs are not completely uniform. In addition, the sample size was small. A larger study should be carried out to confirm this result.

## Author contributions

**Data curation and resources:** Meijuan Song, Xiufang Tian.

**Resources:** Meijuan Song, Xiufang Tian.

**Writing – original draft:** Yajuan Lv.
